# Change in the Green-Up Dates for *Quercus mongolica* in Northeast China and Its Climate-Driven Mechanism from 1962 to 2012

**DOI:** 10.1371/journal.pone.0130516

**Published:** 2015-06-22

**Authors:** Deqin Fan, Wenquan Zhu, Zhoutao Zheng, Donghai Zhang, Yaozhong Pan, Nan Jiang, Xiafei Zhou

**Affiliations:** 1 State Key Laboratory of Earth Surface Processes and Resource Ecology, Beijing Normal University, Beijing 100875, China; 2 College of Resources Science & Technology, Beijing Normal University, Beijing 100875, China; Université du Québec à Chicoutimi, CANADA

## Abstract

The currently available studies on the green-up date were mainly based on ground observations and/or satellite data, and few model simulations integrated with wide coverage satellite data have been reported at large scale over a long time period (i.e., > 30 years). In this study, we combined phenology mechanism model, long-term climate data and synoptic scale remote sensing data to investigate the change in the green-up dates for *Quercus mongolica* over 33 weather stations in Northeast China and its climate-driven mechanism during 1962-2012. The results indicated that the unified phenology model can be well parameterized with the satellite derived green-up dates. The optimal daily mean temperature for chilling effect was between -27°C and 1°C for *Q*. *mongolica* in Northeast China, while the optimal daily mean temperature for forcing effect was above -3°C. The green-up dates for *Q*. *mongolica* across Northeast China showed a delayed latitudinal gradient of 2.699 days degree^-1^, with the earliest date on the Julian day 93 (i.e., 3^th^ April) in the south and the latest date on the Julian day 129 (i.e., 9^th^ May) in the north. The green-up date for *Q*. *mongolica* in Northeast China has advanced 6.6 days (1.3 days decade^-1^) from 1962 to 2012. With the prevailing warming in autumn, winter and spring in Northeast China during the past 51 years, the chilling effect for *Q*. *mongolica* has been weakened, while the forcing effect has been enhanced. The advancing trend in the green-up dates for *Q*. *mongolica* implied that the enhanced forcing effect to accelerate green-up was stronger than the weakened chilling effect to hold back green-up while the changes of both effects were caused by the warming climate.

## Introduction

The response of plant phenology to climate change indicated that the phenological records at large scale and over long time periods may more accurately reflect the variations in climate [1, 2]. For example, the green-up date was mainly driven by temperature for temperate regions at high latitudes [3, 4], while it was mainly controlled by water for semi-arid/sub-humid regions at low latitudes [5]. As a response to global warming, plant phenology shifts can cause changes in community composition and structure, vegetation productivity, as well as exchanges of carbon, water and thermal energy among soil, vegetation and atmosphere [6, 7], and these changes in turn affect the climate system and further accelerate climate change [8].

Model simulation is one of the principal approaches for studying the responses of plant phenology to climate change [4, 9]. Based on the assumption that the budburst was mainly due to the chilling and/or forcing effect, many phenology mechanism models were proposed, such as the Spring Warming model [10], Parallel model [11], Sequential model [11, 12], Alternating model [13], and so on. Chuine summarized these models and proposed a unified model to approximate all of them [9]. This unified model can be simplified according to standard statistical tests for any particular species, and it provides a standardized framework for phenological models, which is essential for comparative studies as well as for robust model identification. Currently, the existing phenology mechanism models were mainly applied for individual plant species [11, 14, 15], rather than for plant population, community or ecosystem. There are two major reasons for the lack of the phenology simulation at large scale. One is the fewer phenology observations at regional scale. Another reason is the increasing number of factors that affect plant phenology with scale-up, including not only environmental factors but also the differences and the interactions among species [16]. These diverse factors make the plant phenology processes more complex and difficult to be simulated at large scale.

Satellite remote sensing is a powerful tool to monitor and characterize the spatial-temporal change in vegetation phenology at the population, community and ecosystem scales [17]. The satellite-derived vegetation phenological metrics are generally generated from vegetation index time-series data, such as the Normalized Difference Vegetation Index (NDVI) and the Enhanced Vegetation Index (EVI) derived from the National Oceanic and Atmospheric Administration (NOAA)/Advanced Very High Resolution Radiometer (AVHRR), SPOT VEGETATION (SPOT-VGT), Moderate Resolution Imaging Spectroradiometer (MODIS) and other sensors. However, these vegetation index time series have a relatively short time span and a coarse spatial resolution. For example, the NOAA/AVHRR Global Inventory Modeling and Mapping Studies (GIMMS) NDVI3g data is currently the longest vegetation index time series. It is just a little more than 30 years with its time spanning from 1982 to now [18]. A coarse spatial resolution (e.g., 8 km for NOAA/AVHRR and 250 m for MODIS data) makes a pixel of the vegetation index time-series data nearly always cover multiple plant species, which makes it difficult to explain the specific climate-driven mechanism in phenology shift at the population scale. Therefore, remote sensing data are limited in their ability to reveal phenology shift and its specific climate-driven mechanism over a much longer period though they can provide a wide coverage scope.

Climate data are usually used to drive the phenology mechanism models, and their records usually go back 50 years or longer [19]. Therefore, combining phenology mechanism models, synoptic remote sensing data and long-term climate data provides opportunities to investigate phenology shift and its specific climate-driven mechanism at large scale over a much longer period. For example, Botta et al. [20] used satellite-derived leaf onset dates and climate data to extrapolate different process-based phenological models established at the stand level, and reconstructed the spatial distribution of the climatological leaf onset date from 1983 to 1993 at the global scale. Delbart et al. [21] used satellite-derived green-up dates to calibrate a green-up mechanism model, and then the model was driven by climate data to analyze phenological variations in Eurasian taiga over nearly a century. However, these studies mainly focus on the spatial-temporal change in vegetation phenology at large scale over long periods, and the climate-driven mechanism in phenology shift (e.g., the contribution of the chilling and forcing effect to green-up) is still absent at the population scale mainly because of the mixture in a coarse satellite pixel with multiple plant species.

Vegetation phenology in Northeast China has shifted during the past 30 years under a warming climate [22]. The start date of the growing season for broadleaf forests in Northeast China has advanced by 2.0 days decade^-1^ from 1982 to 2003, based on investigations using the short-term GIMMS NDVI data [23], while the start date of the thermal growing season has advanced by 1.7 and 1.1 days decade^-1^ based on analyses of the long-term temperature data from 1951 to 2007 [24] and from 1959 to 2008 [25], respectively. The study results based on the short-term remote sensing time series may not be sufficient to reveal the change trend in the vegetation phenology in Northeast China. For example, some studies indicated that the spring vegetation phenology in the Qinghai-Tibet plateau, China and the Northern Hemisphere were advanced based on the time series from 1982 to the 1990s, but then the advancing trends were leveled off or even reversed based on the extended time series from 1982 to the 2000s [1, 26, 27]. Although an advancing trend in the start date of the thermal growing season was observed using long-term climate data, a similar advancing trend in vegetation phenology was not guaranteed because of the differences between vegetation phenology and climate phenology.

Taking the widely distributed *Quercus mongolica* in Northeast China as the study subject, this study aims to combine phenology mechanism model, long-term climate data and synoptic scale remote sensing data to (i) reveal the long-term change trend in the green-up dates for *Q*. *mongolica* during the past 51 years (i.e., 1962–2012), and (ii) analyze the climate-driven mechanism (i.e., the contribution of the chilling and forcing effect to green-up) for the change in the green-up dates.

## Data and Methods

### Study Area

**Fig 1:**
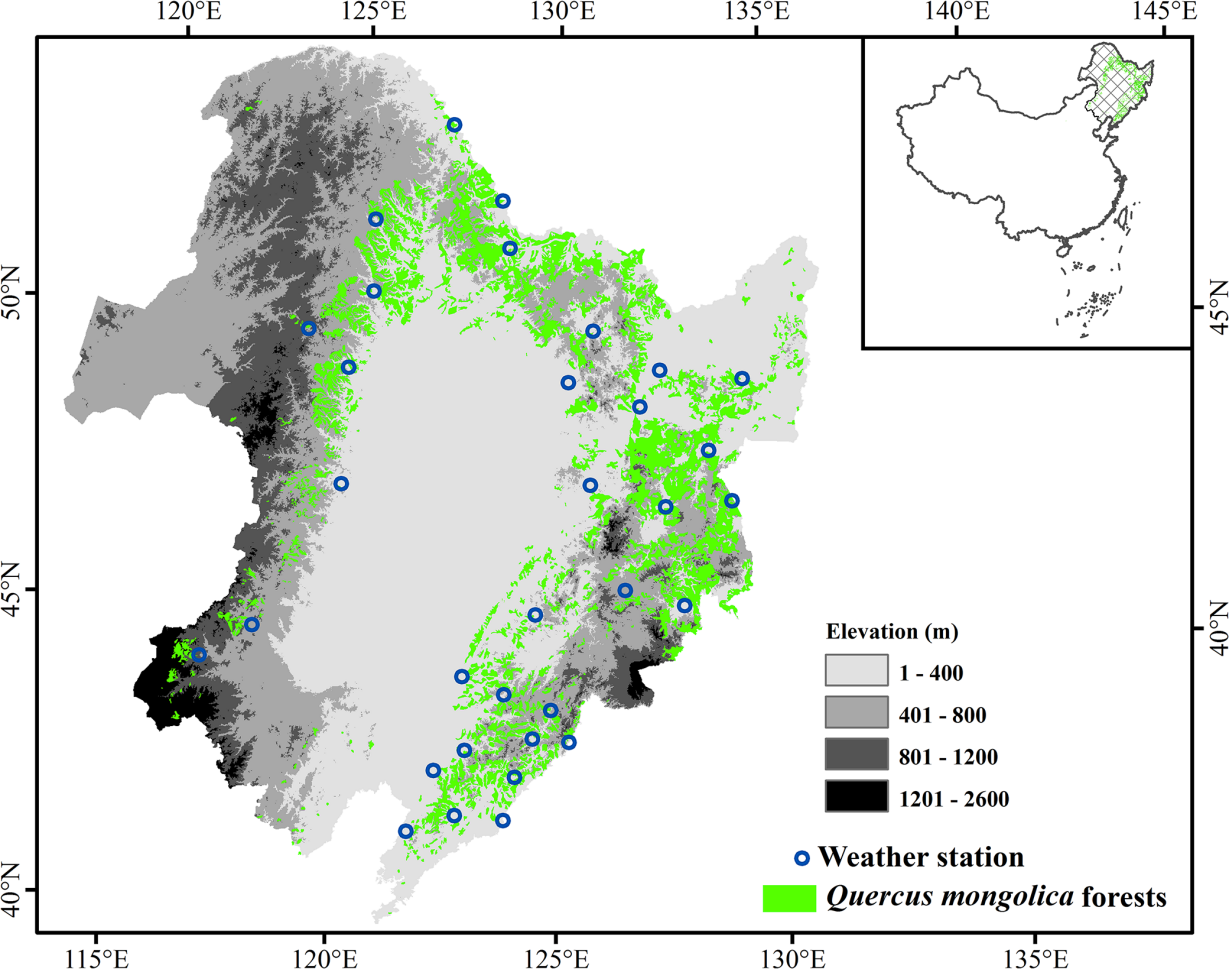
The distribution of *Q*. *mongolica* forests and the weather stations in Northeast China.

Northeast China (38°72′-53°55′N, 115°52′-135°09′E) is characterized by the Northeast China Plain, which is surrounded by mountains (Fig 1). Northeast China has a long and cold winter, with daily mean temperature below -10°C, and a warm, wet and short summer. The mean annual precipitation is approximately 1000 mm in the east and gradually decreases to 400 mm in the west [28]. *Q*. *mongolica* is one of the main broadleaf deciduous forests in Northeast China (Fig 1) [29]. The *Q*. *mongolica* forests are mainly found in the mountains with a low altitude (below 800 m). Green-up for *Q*. *mongolica* usually occurs in May, the flowering date is in June, and the fruit stage is in September [30].

### Data

Climate data at 33 weather stations (Fig 1) were derived from the China Meteorological Data Sharing Service System (http://cdc.nmic.cn/home.do). The daily mean temperature for each station from 1961 to 2012 was used in this study.

The MODIS Land products from the Terra satellite (MOD13Q1, version 005) with a 16-day interval and a 250-meter resolution from 2001 to 2012 were used to derive the green-up dates for *Q*. *mongolica* [31]. The NDVI data in the products were composited using a 16-day maximum value composite method; thus, for each grid cell the NDVI data contained 23 values per year, and the 12-year NDVI time-series data contained 276 values. The NDVI time-series data for each weather station were visually selected based on the 1:1,000,000 vegetation map of China [29] (Fig 1) and the high resolution Google Earth images. Specifically, more than three MODIS NDVI pixels with *Q*. *mongolica* as the dominant species were selected within a 5-km range of each weather station, and then the NDVI values were spatially averaged to represent the NDVI values of each station for further analysis.

### Methods

#### Deriving the Green-up Date based on the NDVI Time Series

The green-up date estimating methods from remote sensing data generally include two processes: reconstructing high-quality vegetation index time-series data through noise removal and computing the green-up date from the reconstructed data. We tested the double-Gaussian, double-Logistic and polynomial functions and found that the double-Gaussian function can get the smallest Root Mean Square Error (RMSE) between the original and the reconstructed NDVI time series for *Q*. *Mongolica* forests (S1 File). The maximum slope threshold method was widely used to derive green-up dates from the NDVI time series and showed a high consistency between the observed and retrieved green-up dates [17, 32]. Therefore, we first used the double-Gaussian function [33] to reconstruct the NDVI time series, and then used the maximum slope threshold method [32] to derive the green-up date from the reconstructed NDVI time series. More details can be found in the S2 File.

#### Simulating the Green-up Date with the Unified Phenology Mechanism Model

The unified phenology mechanism model proposed by Chuine [9] was used to simulate the green-up date for *Q*. *mongolica*. We first used a simulated annealing algorithm [34] to parameterize the unified phenology model based on the NDVI-derived green-up dates and the daily mean temperature data from 2001 to 2006. Then, the internal and external validations for the model were performed using the green-up dates and the daily mean temperature data from 2001 to 2006 and from 2007 to 2012, respectively. Finally, we used the parameterized unified phenology model to simulate the green-up date for *Q*. *mongolica* based on the daily mean temperature data from 1961 to 2012. More details can be found in the S3 File.

## Results

### Model Parameterization

**Fig 2:**
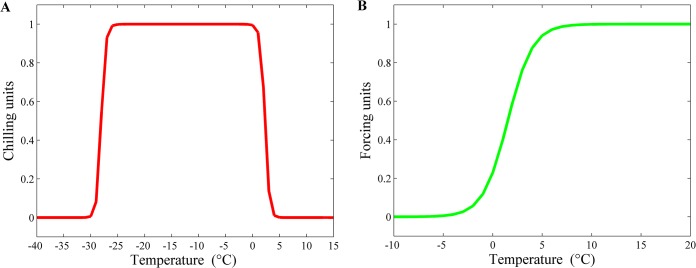
The response functions of (A) the chilling and (B) forcing effect to daily mean temperature for *Q*. *mongolica* in Northeast China.

The optimized parameters for the unified phenology model are presented in Table 1. Based on the optimized parameters in Table 1 and equation S1 and S2 in the S3 File, respectively, the response functions of the chilling and forcing effect to daily mean temperature can be obtained (Fig 2). The chilling effect for *Q*. *mongolica* reached the strongest level when the daily mean temperature was between -27°C and 1°C (Fig 2A). A lower or higher daily mean temperature beyond the optimal chilling temperature range would weaken the chilling effect for the breaking of dormancy. The forcing effect for *Q*. *mongolica* followed a sigmoid curve as the daily mean temperature rose from -7°C to 10°C (Fig 2B). The forcing effect became valid when the daily mean temperature was above -7°C and increased very rapidly when the daily mean temperature rose from -3°C to 5°C.

**Table 1:** The optimized phenology model parameters for *Q*. *mongolica*.

*a*_*c*_	*b*_*c*_	*c*_*c*_	*b*_*f*_	*c*_*f*_	*w*	*k*	*C* ^*^	*t*_*c*_
0.0828	-2.515	-28.072	-0.796	1.5287	37.109	-0.00279	18.322	201.959

### Model Validation

**Fig 3:**
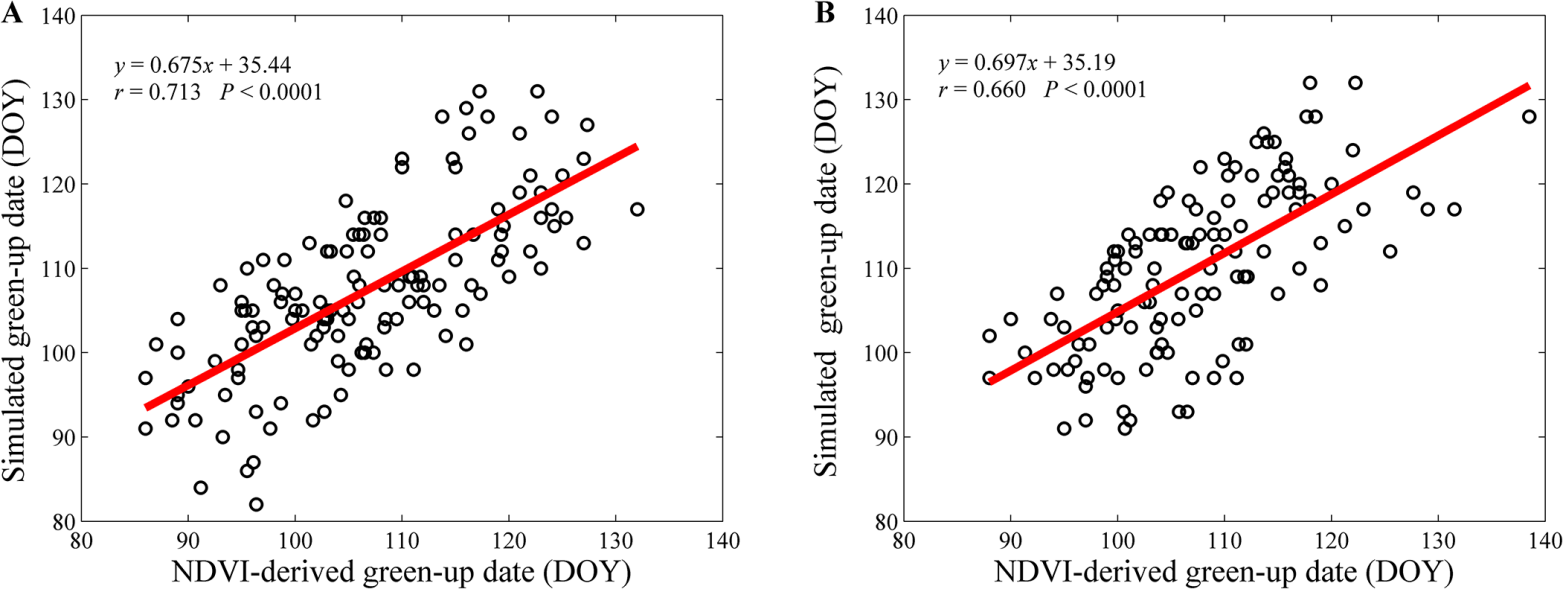
The (A) internal and (B) external validations for the green-up dates of *Q. mongolica* forests simulated by the unified phenology model.

We validated the simulated green-up dates using the NDVI-derived green-up dates (Fig 3). The correlation coefficient and RMSE between the simulated and the NDVI-derived green-up dates were 0.713 (*P* < 0.001) and 7.08 days (Fig 3A), respectively, if the same data as model parameterization (i.e., daily mean temperature and NDVI data from 2001 to 2006; internal validation) were used in the evaluation, and 0.660 (*P* < 0.001) and 7.30 days (Fig 3B), respectively, if the different data from model parameterization (i.e., daily mean temperature and NDVI data from 2007 to 2012; external validation) were used.

### The Simulated Green-up Date for *Q*. *mongolica*

**Fig 4:**
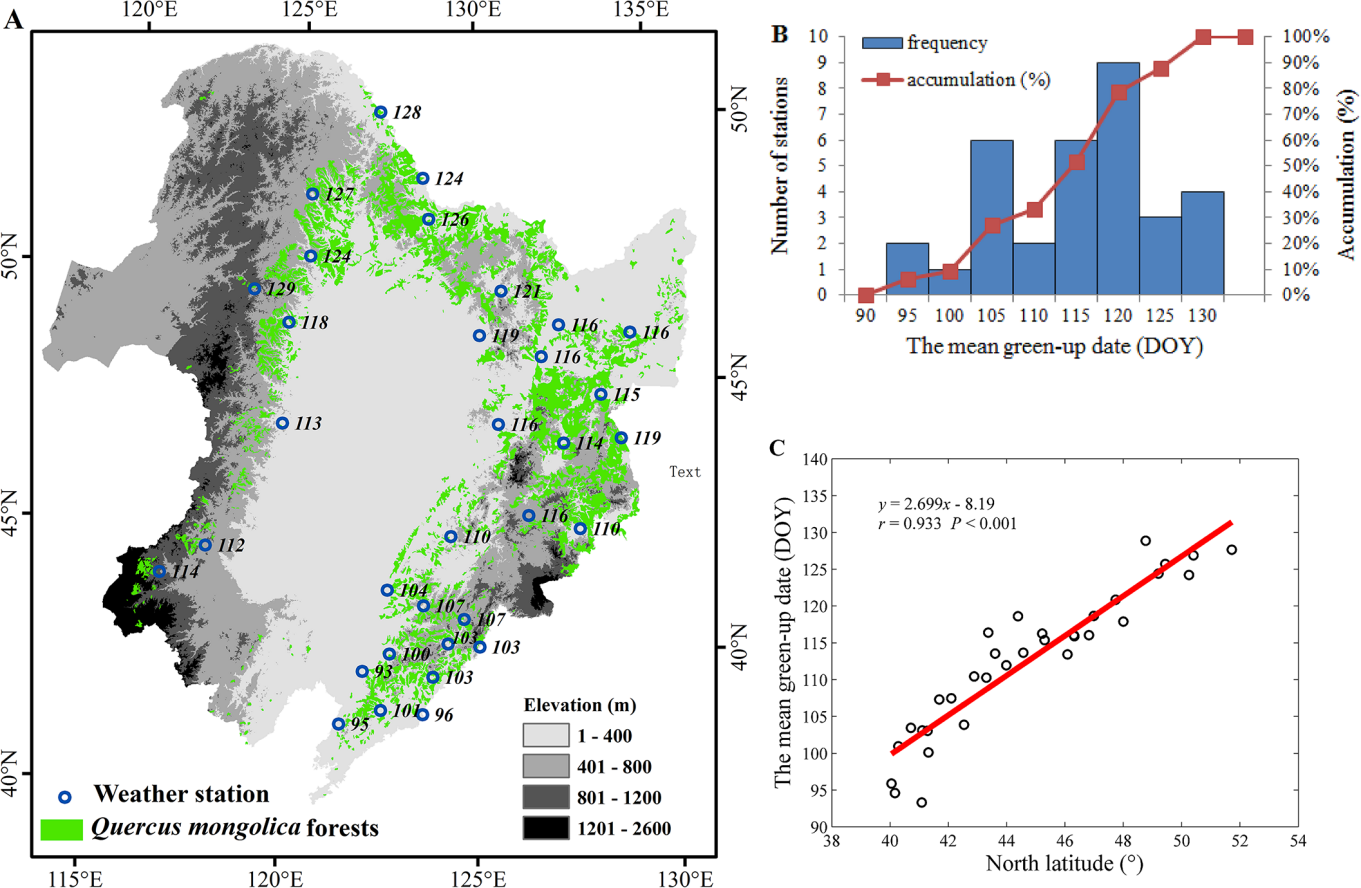
The temporal mean green-up dates (DOY) for *Q*. *mongolica* in Northeast China during 1962–2012. (A) The spatial distribution of mean green-up dates over 33 weather stations; (B) the frequency distribution of green-up dates; and (C) the relationship between the green-up date and latitude. Note: the numbers in (A) indicate the green-up dates (DOY) at each weather station.

Fig 4A shows the spatial distribution of the temporal mean green-up dates for *Q*. *mongolica* during 1962–2012. The mean green-up dates mainly occurred between DOY 93 (i.e., 3^th^ April) and 129 (i.e., 9^th^ May) across all 33 stations (Fig 4B). As the latitude increases toward the north, the green-up date was delayed at a rate of 2.699 days degree^-1^ (Fig 4C). The significant relationship (*P* < 0.001) between green-up date and latitude reflected the primary control of temperature over green-up date.

**Fig 5:**
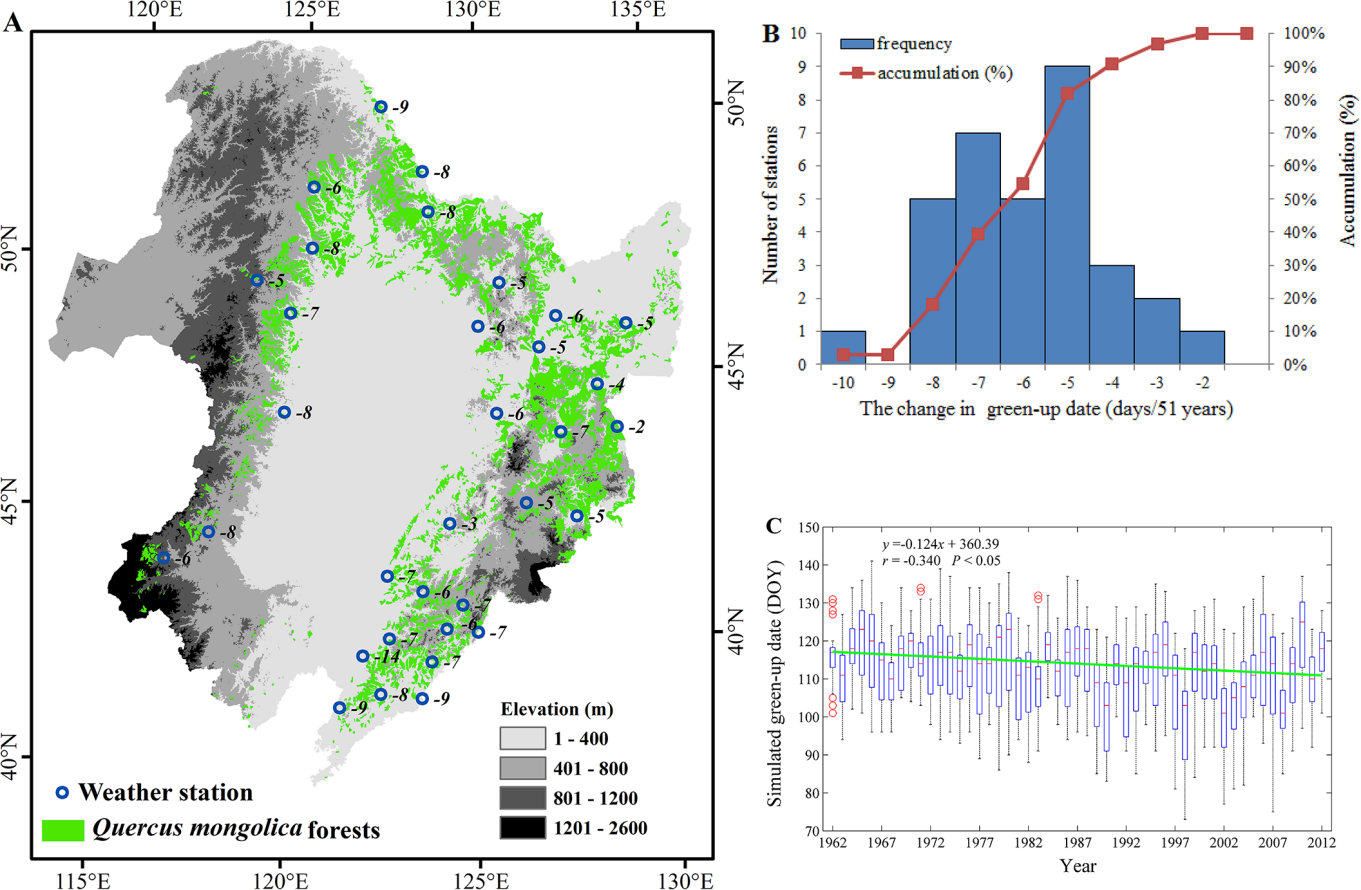
The temporal changes in the green-up dates for *Q*. *mongolica* in Northeast China from 1962 to 2012. (A) The temporal changes in the green-up dates over 33 weather stations; (B) the frequency distribution of the temporal changes in green-up dates; and (C) the interannual variations in green-up dates (DOY) for the 33 stations and the change trend (days decade^-1^). Note: the numbers in (A) indicate the advanced (minus number) or delayed (plus number) days at each weather station.

The temporal changes in the green-up dates are shown in Fig 5. The *Q*. *mongolica* forests across all the 33 stations in Northeast China exhibited an advancing trend in the green-up dates during 1962–2012 (Fig 5A and 5B)). The average advancing trend for the 33 stations was 1.3 days decade^-1^ (i.e., advanced 6.6 days from 1962 to 2012) (Fig 5C).

## Discussion

### The Reliability and Utility of the Model Simulation

We combined phenology mechanism model, long-term climate data and synoptic scale remote sensing data to investigate the change in the green-up dates for *Q*. *mongolica* in Northeast China from 1962 to 2012 and its climate-driven mechanism. The chilling and forcing effect computed from the parameterized unified phenology model with the satellite-derived green-up dates were comparable to others with ground-observed green-up dates [9, 35]. Our results indicated that the chilling effect for *Q*. *mongolica* reached the strongest level when the daily mean temperature was between -27°C and 1°C, while the forcing effect followed a sigmoid curve as the daily mean temperature rose from -3°C to 10°C. Wang *et al*. reported that the chilling effect computed from the parameterized unified phenology model with ground-observed green-up dates for *Fraxinus chinensis* in China reached the strongest level when the daily mean temperature was between -35°C and 20°C, and its forcing effect also followed a sigmoid curve as the daily mean temperature rises from 0°C to 40°C [35]. Other studies based on ground-observed green-up dates also indicated that the chilling effect for *Aesculus hippocastanum*, *Buxus sempervirens*, *Olea europaea* and *Ulmus minor* reached the strongest level when the daily mean temperature were between -40°C and -10°C, -5°C and -3°C, -23°C and -10°C, and -3°C and 8°C, respectively [9], and the forcing effect for them all followed a sigmoid curve as the daily mean temperature rose from -12°C to -8°C, -15°C to -8°C, -30°C to 0°C, and -15°C to 0°C, respectively [9]. These comparable chilling and forcing effects computed from the parameterized unified phenology model with different data sources (i.e., satellite-derived vs. ground-observed green-up dates) indicated that the unified phenology model can be well parameterized with the satellite-derived green-up dates.

The RMSEs for the internal and external validation were 7.08 and 7.30 days, respectively, which were similar to the validation results based on the ground observed data from spring phenophase of *Fraxinus chinensis* in China during 1952–2007 (the RMSEs for both internal and external validations were 6.1 days) [35]. This similarity indicated that the simulation accuracy for the unified phenology model at the population level based on NDVI data can be comparable to that based on the ground observations at the individual species level.

Combining phenology mechanism model, long-term climate data and synoptic scale remote sensing data to investigate phenology shift and its climate-driven mechanism has wide utilities. First, this approach can be extended to other species if they are very dominant species and cover a large area. For example, a single crop species is usually largely distributed in plains (e.g., winter wheat in North China Plain). So we can use the satellite-derived crop phenological metrics [36] and climate data to parameterize the unified phenology model. Second, the phenology mechanism model parameterized with satellite-derived phenological metrics and climate data can be further integrated with terrestrial ecosystem processing models to study the impacts of climate change on ecosystem composition, structure and function [37, 38]. The parameterized phenology mechanism model can be also integrated with crop models to simulate crop growth process and forecast crop yields [39, 40]. However, we should still note that the reliability of the unified phenology model parameterized with the satellite-derived phenological metrics may be affected by multiple factors, such as the quality and spatial resolution of the remote sensing data and the vegetation distribution characteristics (e.g., purity or mixture). Therefore, the time-series satellite data with high quality and high spatial resolution is preferred. Fortunately, the amount of such high quality satellite time-series data has increased in recent years and will continue to increase in the future. For example, the small environmental monitoring satellite constellation (Environment-1) in China can obtain 30-meter resolution images with a 2- to 3-day revisit cycle, and the European Sentinel-2 can obtain 10- to 60-meter resolution images with a 5-day revisit cycle.

### The Climate-driven Mechanism in the Change of Green-up Date for *Q*. *mongolica*

Temperature accumulations are required to fulfill both the chilling and forcing effect for the green-up of *Q*. *mongolica*. A prevailing warming in autumn, winter and spring was observed in Northeast China during the past 51 years (S4 File). The daily mean temperature during the chilling period has increased by 0.41°C decade^-1^, and the daily mean temperature during the forcing period has increased by 0.26°C decade^-1^ in Northeast China from 1962 to 2012 (S4 File). The number of days with an optimal chilling temperature (i.e., between -27°C and 1°C) during the chilling period had no significant change, meanwhile, the number of days with an effective forcing temperature (i.e., above -7°C) during the forcing period also had no significant change from 1962 to 2012 (S4 File). These changes in temperature implied that the reduced chilling effect has held back green-up, while the enhanced forcing effect has accelerated green-up for *Q*. *mongolica* forests. Morin et al. [41] used a simplified version of the unified phenology model to predict the leaf phenology in 22 North American tree species during the 21^st^ century. Their results indicated that climate change would affect leaf phenology in almost all studied species, with average advancements of 5.0 days and 9.2 days, when the global mean temperature increased 3.2°C and 1°C, respectively, during the 21^st^ century. They suggested that lack of sufficient chilling temperatures to break bud dormancy may decrease the rate of advancement in green-up date during the 21^st^ century for many species. Therefore, with the increase in temperature in autumn, winter and spring in Northeast China, the insufficient chilling requirement may be far exceeded by the enhanced forcing effect, which therefore resulted in an advancing trend in the green-up date for *Q*. *mongolica* in Northeast China during 1962–2012.

### The Spatio-temporal Change Trend in the Green-up Dates for *Q*. *mongolica*

The green-up dates for *Q*. *mongolica* across Northeast China showed a significant latitudinal gradient with the earliest date on the Julian day 93 (i.e., 3^th^ April) in the south and the latest date on the Julian day 129 (i.e., 9^th^ May) in the north. As the latitude increases toward the north, the green-up date was delayed at a rate of 2.699 days degree^-1^. Since the NDVI value for each weather station in one year was the spatial mean of 3–5 visually selected pixels within a 5-kilometer range of the weather station, the variation among the pixels within a weather station may affect the spatial distribution of green-up date across Northeast China (i.e., the spatial variation among weather stations). Actually, the standard deviation in green-up date among the selected pixels around the weather station within a year was about 4 days (S5 File), while the spatial variation among weather stations was 36 days (Fig 4C). So we can conclude that the spatial distribution of green-up date across Northeast China was mainly caused by the spatial differences among weather stations but not the variation among pixels within a weather station. Moreover, Li et al. [42] indicated that the green-up date of woody plants was delayed at a rate of 3.0–4.0 days degree^-1^ with the latitude increases toward the north from 1980 to 2005 based on ground observations. Chen et al. [43] demonstrated that the green-up date was delayed at a rate of 2.7–4.0 days degree^-1^ with the latitude increases toward the north in the north-subtropical zone of eastern China from 1982 to 2006.

Our simulated results indicated that the green-up date for *Q*. *mongolica* across 33 weather stations in Northeast China has advanced 6.6 days from 1962 to 2012 (i.e., 1.3 days decade^-1^). Guo et al. [23] demonstrated that the start date of the growing season for the broadleaf forest in Northeast China has advanced 0.6 days decade^-1^ using the GIMMS NDVI time-series data from 1982 to 2003, while When focusing on the same periods, the simulated green-up date for *Q*. *mongolica* has advanced 3.1 days decade^-1^ from 1982 to 2003, and 2.5 days decade^-1^ from 1980 to 2005. The above comparisons from different data sources (i.e., ground observations, satellite data and simulated results) all indicated that the green-up date in Northeast China has exhibited an advanced trend since the 1980s. Moreover, the advanced trend in the green-up dates for *Q*. *mongolica* was also highly consistent with that in the start dates of the thermal growing season (S6 File), which further proved the advanced trend in the green-up dates for *Q*. *mongolica* in Northeast China during 1962–2012.

## Conclusions

In this study, we investigated the spatio-temporal change in the green-up dates for *Q*. *mongolica* in Northeast China from 1962 to 2012 and its climate-driven mechanism through combining the unified phenology model, long-term climate data and synoptic scale remote sensing data. The results indicated that the optimal chilling daily mean temperature for *Q*. *mongolica* in Northeast China was between -27°C and 1°C, while the forcing effect became effective when the daily mean temperature was above -7°C with the optimal forcing temperature above -3°C. The green-up dates mainly occurred between the Julian day 93 (i.e., 3^th^ April) and 129 (i.e., 9^th^ May) across Northeast China. As the latitude increases toward the north, the green-up date was delayed at a rate of 2.699 days degree^-1^. An advancement of 6.6 days was observed in the green-up date for *Q*. *mongolica* in Northeast China from 1962 to 2012. Due to the prevailing warming in autumn, winter and spring in Northeast China during the past 51 years, the chilling effect for *Q*. *mongolica* has been weakened, while the forcing effect has been enhanced. Therefore, the advancing trend in the green-up date for *Q*. *mongolica* in Northeast China during the past 51 years was mainly attributed to the enhanced forcing effect from the warming winter and spring.

This study demonstrated the investigation on phenology shift and its climate-driven mechanism at large scales over a much longer period through the combination of phenology mechanism model, long-term climate data and synoptic scale remote sensing data. Establishing this link can provide a potentially powerful tool to extend the relatively limited ground observations to the spatial domain covered by remote sensing observations and to extend the short-term remote sensing data to a long time series as instrument observed climate records, thereby providing a comprehensive view of the phenology shift and its climate-driven mechanism.

## Supporting Information

S1 File Comparison of Different NDVI Reconstructing methods. (DOCX)

S2 File Deriving the Green-up Date based on the NDVI Time Series. (DOCX)

S3 File Simulating the Green-up Date with the Unified Phenology Mechanism Model. (DOCX)

S4 File Climate Change in Northeast China during 1962–2012. (DOCX)

S5 File The Variation in Green-up Dates among the Pixels within a Weather Station. (DOCX)

S6 File The Relationship between Simulated Green-up Dates for *Q*. *mongolica* and the Start Dates of the Thermal Growing Season. (DOCX)
