# Supplementary material for: Change in the Green-Up Dates for Quercus mongolica in Northeast China and Its Climate-Driven Mechanism from 1962 to 2012
Source: PLoS One. 2015 Jun 22;10(6):e0130516. doi: 10.1371/journal.pone.0130516 (PMC4476677; doi:10.1371/journal.pone.0130516)
Supplement: S1 File — (DOCX) [file pone.0130516.s001.docx]

**S1 File. Comparison of Different NDVI Reconstructing methods**

Among the double-Gaussian, double-Logistic and polynomial functions, the double-Gaussian function was the best fitting method to describe the intra-annual growth cycle for Kobresia pygmaea alpine meadow [1]. The reconstructed Normalized Difference Vegetation Index (NDVI) time series with the double-Gaussian function had the smallest deviation (RMSE) from the original NDVI time series, and the noises can be reduced effectively during the double-Gaussian fitting process (Fig. S1) [1]. Considering both the *Q. mongolica* forests and the K. pygmaea alpine meadow have only one growing season in a year and both are distributed at the same temperate zone, so we selected the double-Gaussian function to reconstruct the NDVI time series for *Q. Mongolica* forests in this paper.

Fig. S1. **The RMSEs for the three NDVI fitting methods from 1991 to 2010**

**References**

[1] Fan DQ, Zhu WQ, Pan YZ, Jiang N, Zheng ZT. Identifying an optimal method for estimating greenup date of *Kobresia pygmaea* alpine meadow in Qinghai-Tibetan Plateau. J. Remote Sens. 2014; 18(5): 1117-1127.
