# Supplementary material for: Change in the Green-Up Dates for Quercus mongolica in Northeast China and Its Climate-Driven Mechanism from 1962 to 2012
Source: PLoS One. 2015 Jun 22;10(6):e0130516. doi: 10.1371/journal.pone.0130516 (PMC4476677; doi:10.1371/journal.pone.0130516)
Supplement: S2 File — (DOCX) [file pone.0130516.s002.docx]

**S2 File. Deriving the Green-up Date based on the NDVI Time Series**

The green-up date estimating methods from remote sensing data generally include two processes: reconstructing high-quality vegetation index time-series data through noise removal and computing the green-up date from the reconstructed data.

Reconstructing the NDVI time series with the double-Gaussian function fitting method

NDVI time-series data derived from middle- or low-resolution sensors contain a large amount of noise caused by the solar altitude angle, satellite observation angle, cloud coverage, water vapor content and aerosol content. The noise obscures the seasonal changes and the phenology characteristics in the NDVI time series, which hinders the identification of the green-up date. Function fitting is a widely used method to reconstruct NDVI time-series data [1, 2]. We chose the Gaussian function system (equation S1) [3] to remove noise in the original NDVI time-series data.

| **$g\left(t\right)=\sum_{i=1}^{n} a_{i}\times\exp[-\left(\frac{t-b_{i}}{c_{i}}\right)^{2}]$** | (S1) |
| --- | --- |

where the peak height (*a_i_*), peak position (*b_i_*) and peak width (*c_i_*) are three parameters of the Gaussian function system, *n* is the number of Gaussian functions, and *t* indicates the Julian day of year (DOY). Because *Q. mongolica* forests in Northeast China have only one growing season in a year, we can use a double-Gaussian function (two Gaussian functions when setting *n* to 2) to fit the NDVI time series. The six unknown parameters (*a_1_*, *b*_1_, *c*_1_, *a*_2_, *b*_2_, *c*_2_) can be determined by a global optimization based on a simulated annealing algorithm [4].

Deriving the green-up date with the maximum slope threshold method

The maximum slope threshold method [5, 6] was chosen to derive the green-up date from the NDVI time-series data. In this study, the 16-day interval multi-year average NDVI time-series curve (i.e., 23 data points for a year) from 2001 to 2012 for each pixel was first computed and then linearly interpolated to a daily time series (i.e., 365 data points for a year). The maximum slope NDVI_max__*_ratio(t)_* in the daily NDVI time-series curve can be then derived with equation S2:

|  | (S2) |
| --- | --- |

where *t* (*t* =1,2,3,…365) indicates DOY . For each year, the green-up date can be determined when its NDVI slope reaches to the threshold NDVI_max__*_ratio_*_(_*_t_*_)_.

**References**

[1] Beck PSA, Atzberger C, Hogda KA, Johansen B, Skidmore AK. Improved monitoring of vegetation dynamics at very high latitudes: a new method using MODIS NDVI. Remote Sens. Environ. 2006; 100(3): 321-334. doi: 10.1016/j.rse.2005.10.021.

[2] Jönsson P, Eklundh L. TIMESAT-a program for analyzing time-series of satellite sensor data. Comput Geosci-UK. 2004; 8(30): 833-845. doi: 10.1016/j.cageo.2004.05.006.

[3] Li M, Sheng Y. Study on application of Gaussian fitting algorithm to building model of spectral analysis. Spectrosc Spect Anal. 2008; 28(10): 2352-2355.

[4] Kirkpatrick S, Gelatt CD, Vecchi MP. Optimization by simulated annealing. Science. 1983; 220(4598): 671-680. doi: 10.1126/science.220.4598.671.

[5] Zhang GL, Zhang YJ, Dong JW, Xiao XM. Green-up dates in the Tibetan Plateau have continuously advanced from 1982 to 2011. Proc. Natl. Acad. Sci. USA. 2013; 110(11): 4309-4314. doi: 10.1073/pnas.1210423110.

[6] Piao SL, Fang JY, Zhou LM, Ciais P, Zhu B. Variations in satellite-derived phenology in China's temperate vegetation. Glob. Change Biol. 2006; 12(4): 672-685. doi: 10.1111/j.1365-2486.2006.01123.x.
