# Supplementary material for: Change in the Green-Up Dates for Quercus mongolica in Northeast China and Its Climate-Driven Mechanism from 1962 to 2012
Source: PLoS One. 2015 Jun 22;10(6):e0130516. doi: 10.1371/journal.pone.0130516 (PMC4476677; doi:10.1371/journal.pone.0130516)
Supplement: S3 File — (DOCX) [file pone.0130516.s003.docx]

**S3 File. Simulating the Green-up Date with the Unified Phenology Mechanism Model**

Model principle

The unified phenology mechanism model is based on the accumulations of chilling and forcing temperature [1]. The chilling unit, forcing unit, chilling accumulated temperature and forcing accumulated temperature are expressed in equation S1 to S4.

| $R_{c}=\frac{1}{\text{1+}e^{a_{c}\left(x_{t}\text{-c}_{c}\right)^{2}\text{+}b_{c}\left(x_{t}\text{-}c_{c}\right)}}$ | (S1) |
| --- | --- |
| $R_{f}=\frac{1}{\text{1+}e^{b_{f}\left(x_{t}\text{-}c_{f}\right)}}$ | (S2) |
| $C^{*}\text{=}\sum_{t_{0}}^{t_{1}} R_{c}\left(x_{t}\right)$ | (S3) |
| $F^{*}\text{=}\sum_{t_{1}}^{t_{b}} R_{f}\left(x_{t}\right)$ | (S4) |

where *R_c_* is the chilling unit, *R_f_* is the forcing unit, *C^*^* is the accumulated chilling temperature, *F*^*^ is the accumulated forcing temperature during the forcing period (from *t*_1_ to *t_b_*), *x_t_* is the daily mean temperature, *t_0_* is the start date of dormancy, set as the Julian date on 1^st^ September in the previous year, *t*_1_ is the start date for the forcing units, *t_b_* is the green-up date, *a_c_*, *b_c_* and *c_c_* are the coefficients of the chilling unit function, and *a_f_* and *c_f_* represent the coefficients of the forcing unit function. *t*_0_ can be fixed to 1^st^ September in the previous year because chilling effect does not occur before this time in the temperate zone and buds have been already dormant at the end of summer [1, 2].

The negative relationship between the state of the forcing temperature and the state of the chilling temperature can be described as equation S5. The critical state of forcing required for green-up (*F**) can be described as a function of the total state of achieved chilling (*C_tot_*) as follows:

| $F^{*}=we^{kC_{tot}}$ | (S5) |
| --- | --- |

where *w* > 0 and *k* < 0, $C_{tot}=\sum_{t_{0}}^{t_{c}} R_{c}$ is the accumulated chilling temperature during the chilling period (from *t*_0_ to *t_c_*) and *t_c_* is the end date of the chilling unit. In general, the unified model contains nine parameters [*a_c_*, *b_c_*, *c_c_*, *b_f_*, *c_f_*, *w*, *k*, *C^*^*, *t_c_*] which are needed to be optimized based on the NDVI-derived green-up dates.

Model Parameterization

A simulated annealing algorithm was used to optimize the nine parameters in the unified phenology model [3]. The object function for the simulated annealing algorithm was expressed as equation S6:

| $f\left(x\right)=\sum_{i=1}^{n} \left[r_{i}\left(x\right)\right]^{2}$ | (S6) |
| --- | --- |

where *r_i_*(*x*) is the residual error, *r_i_*(*x*) = *d_i_*(*x*) - *d_iobs_*, *d_i_*(*x*) is the predicted date and *d_iobs_* is the NDVI-derived green-up date in the year *i*.

Model Validation

The NDVI-derived green-up dates during 2001-2012 were divided into two parts. The dates for the first six years (2001-2006) were used for model parameterization and internal validation (i.e., the same dates were used for both model parameterization and model validation), while the other dates for the next six years (2007-2012) were used for the external validation (i.e., the different date were used for model parameterization and model validation). The correlation coefficient and RMSE between the NDVI-derived green-up dates and the model simulated ones were computed to measure the reliability of the parameterized phenology model.

Model Simulation

The daily mean temperature data for each weather station from 1961 to 2012 were used to drive the parameterized phenology model. Therefore, we can simulate the green-up dates for *Q. mongolica* in Northeast China and analyze the change trends in the green-up dates in the past 51 years.

**References**

[1] Chuine I. A unified model for budburst of trees. J. Theor Biol. 2000; 207(3): 337-347. doi: 10.1006/jtbi.2000.2178.

[2] Wang HJ, Dai JH, Ge QS. The spatiotemporal characteristics of spring phenophase changes of *Fraxinus chinensis* in China from 1952 to 2007. Sci China Ser D-Earth Sci. 2012; 55(6): 991-1000. doi: 10.1007/s11430-011-4349-0.

[3] Kirkpatrick S, Gelatt CD, Vecchi MP. Optimization by simulated annealing. Science. 1983; 220(4598): 671-680. doi: 10.1126/science.220.4598.671.
