# Supplementary material for: Change in the Green-Up Dates for Quercus mongolica in Northeast China and Its Climate-Driven Mechanism from 1962 to 2012
Source: PLoS One. 2015 Jun 22;10(6):e0130516. doi: 10.1371/journal.pone.0130516 (PMC4476677; doi:10.1371/journal.pone.0130516)
Supplement: S4 File — (DOCX) [file pone.0130516.s004.docx]

**S4 File. Climate change in Northeast China during 1962-2012**

The daily mean temperature has increased by 0.41°C decade^-1^ during the chilling period (Fig. S1(A)), and 0.26°C decade^-1^ during the forcing period (Fig. S1(B)) in Northeast China from 1962 to 2012. The number of days with an optimal chilling temperature (i.e., -27°C to 1°C) during the chilling period had no significant change (Fig. S2(A)), meanwhile, the number of days with an effective forcing temperature (i.e., above -7°C) during the forcing period also had no significant change from 1962 to 2012 (Fig. S2(B)).


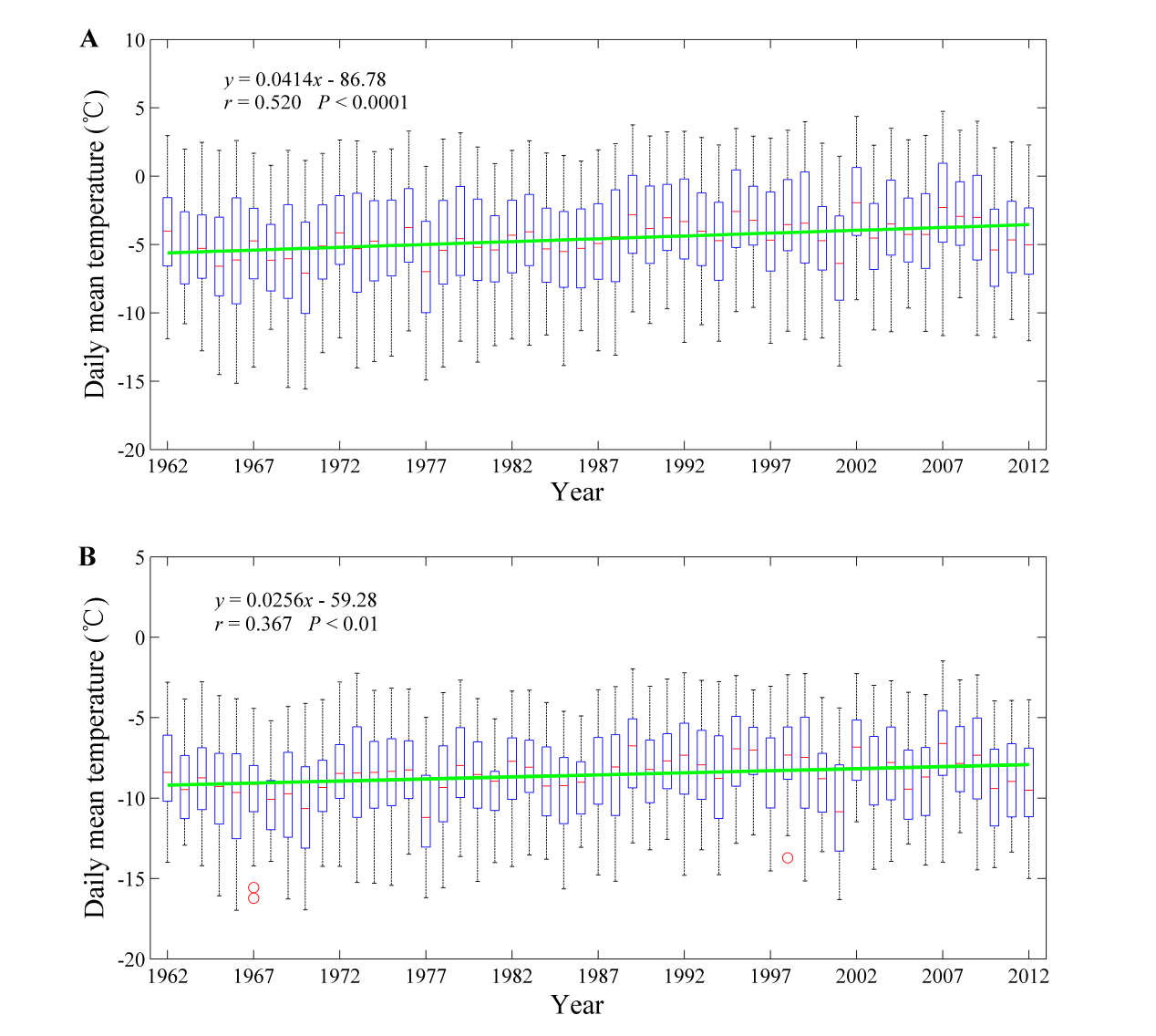


Fig. S1. The daily mean temperature across 33 weather stations in Northeast China and its change trend during 1962-2012 for (A) the chilling period and (B) the forcing period.


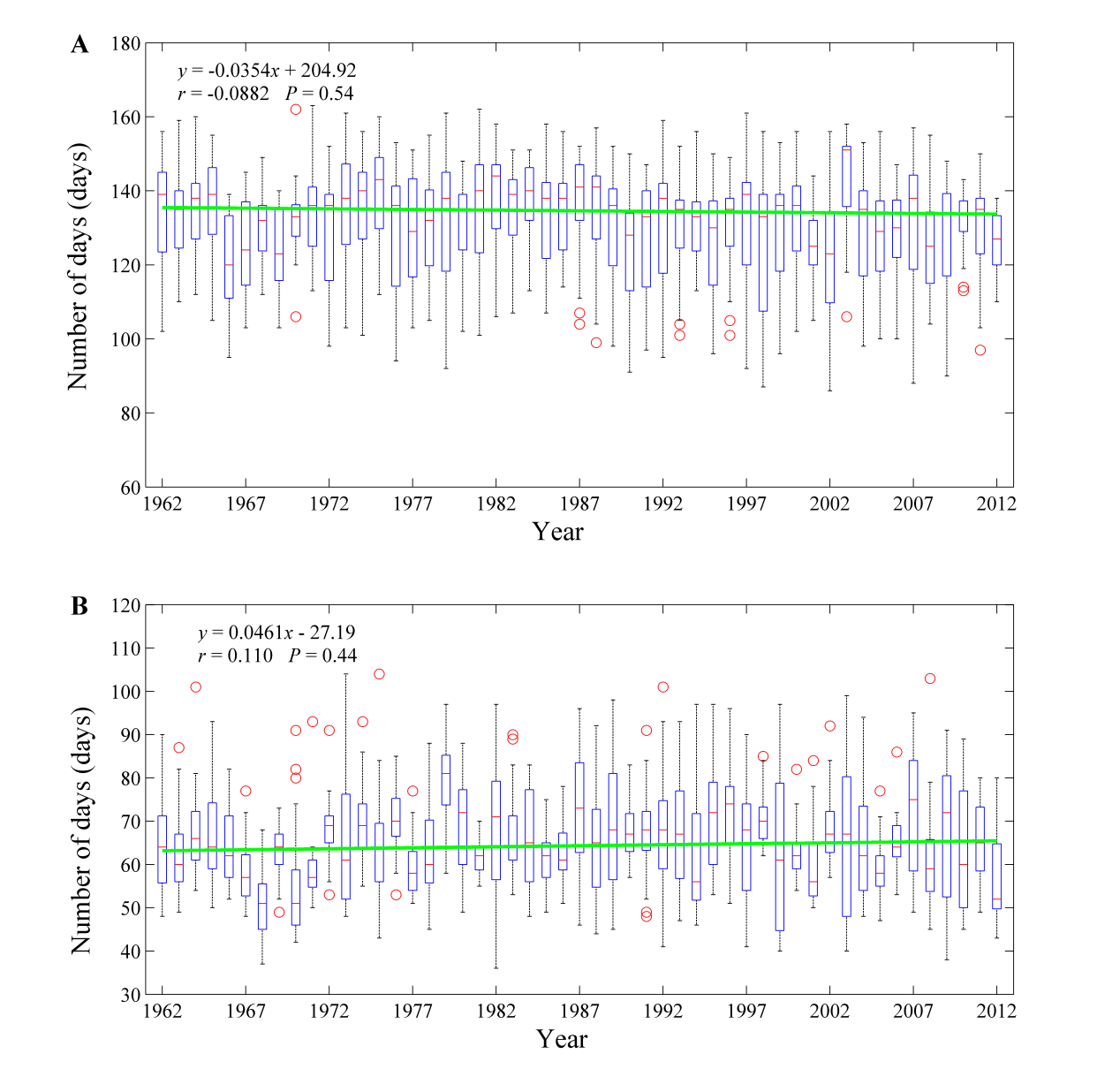


**Fig. S2. The number of days and its change trend during 1962-2012 with (A) an optimal chilling temperature (-27°C to 1°C) during the chilling period and (B) an effective forcing temperature (above -7°C) during the forcing period.**
