# Supplementary material for: Change in the Green-Up Dates for Quercus mongolica in Northeast China and Its Climate-Driven Mechanism from 1962 to 2012
Source: PLoS One. 2015 Jun 22;10(6):e0130516. doi: 10.1371/journal.pone.0130516 (PMC4476677; doi:10.1371/journal.pone.0130516)
Supplement: S5 File — (DOCX) [file pone.0130516.s005.docx]

**S5 File. The Variation in Green-up Dates among the Pixels within a Weather Station**

Since the NDVI value for each weather station in one year was the spatial mean of 3-5 visually selected pixels within a 5-kilometer range of the weather station, the variation among the pixels within a weather station may affect the spatial distribution of green-up date across Northeast China (i.e., the spatial variation among weather stations). In order to make it clear, we analyzed the dispersion in green-up dates among pixels (3 to 5 pixels) within a year around the same weather station (e.g., Tieli weather station (46°59′N, 128°01′E)) from 2001 to 2012. The results indicated that the standard deviation in green-up date among the selected pixels around the weather station within a year was about 4 days (Fig. S1).

**Fig. S1.** **The standard deviation of green-up date among pixels within a 5-kilometer range of the Tieli weather station for different years**
