# Supplementary material for: Change in the Green-Up Dates for Quercus mongolica in Northeast China and Its Climate-Driven Mechanism from 1962 to 2012
Source: PLoS One. 2015 Jun 22;10(6):e0130516. doi: 10.1371/journal.pone.0130516 (PMC4476677; doi:10.1371/journal.pone.0130516)
Supplement: S6 File — (DOCX) [file pone.0130516.s006.docx]

**S6 File. The Relationship between Simulated Green-up Dates for *Q. mongolica* and the Start Dates of the Thermal Growing Season**

Although different environmental limits, such as temperature thresholds, light and water availability, must be considered in investigating vegetation phenology shift in a particular area, the climatological growing season was often defined from temperature alone because temperature was usually the main driver for vegetation phenology shift [1, 2]. For temperate regions, the start date of the climatic (or thermal) growing season was defined as the last day of the first 6-day period when the daily mean temperature was greater than 5°C after the last spring frost (daily mean temperature > 0°C) [3]. We calculated the start date of the thermal growing season after using the 5-day moving average to reduce the effects of sudden changes in the original daily mean temperature data from 1961 to 2012.

The start date of the thermal growing season in Northeast China showed an advanced trend across all the 33 weather stations from 1962 to 2012 (Fig. S1(A)). The average advanced trend for the 33 weather stations was 1.14 days decade^-1^ (i.e., advanced 5.69 days from 1962 to 2012; Fig. S1(B)). The green-up date for *Q. mongolica* was significantly correlated with the start date of the thermal growing season at all stations (Fig. S2), which further substantiated the advanced trend in the green-up dates for *Q. mongolica* in Northeast China from 1962 to 2012.


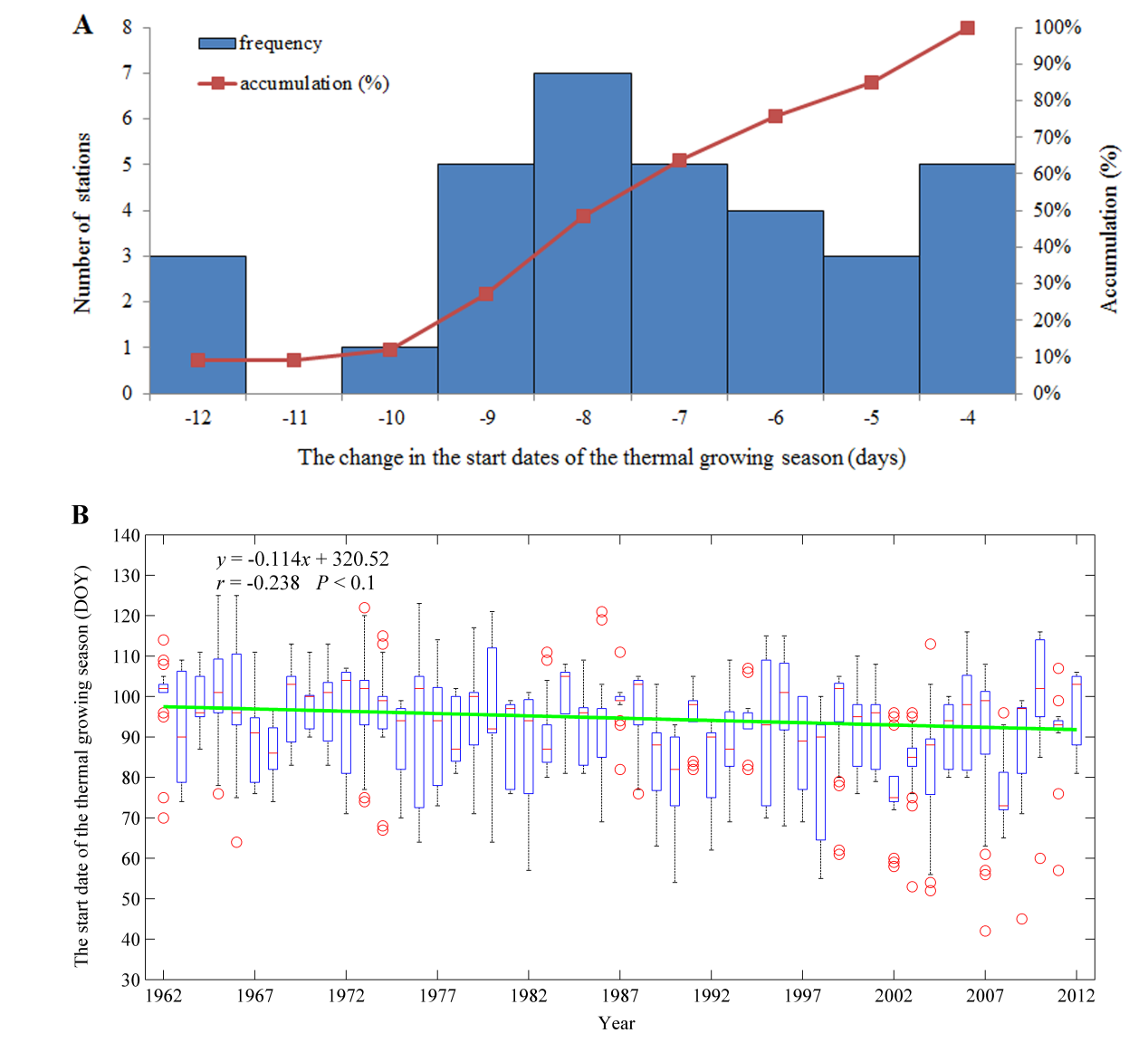


Fig. S1. The change in the start dates of the thermal growing season in Northeast China from 1962 to 2012. (A) Frequency distribution of weather station numbers for different changes; and (B) the box plot of the start dates across 33 weather stations for each year and the change trend in the start dates. Negative values in the *x* axis indicate advanced days.


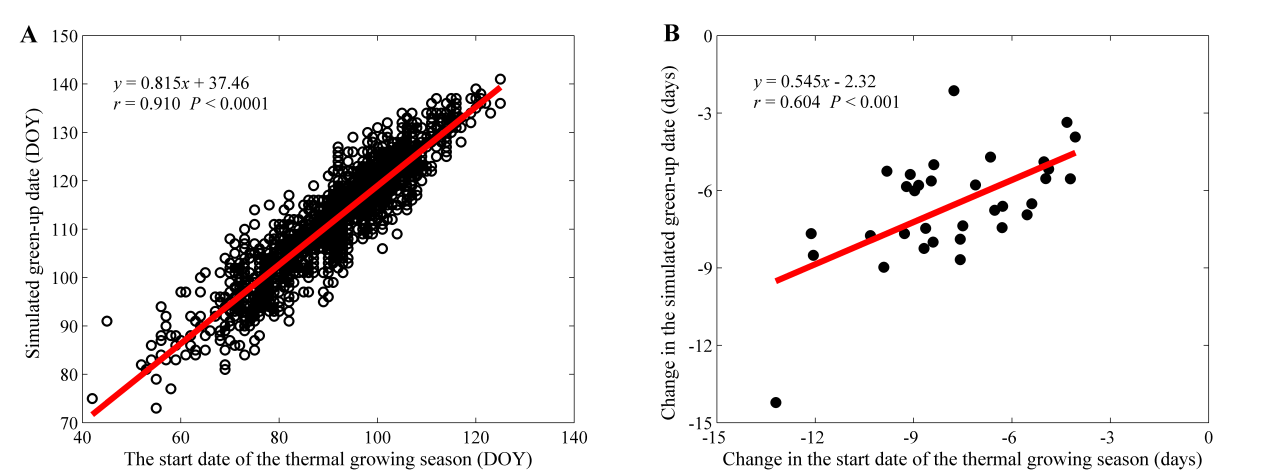


**Fig. S2.** **The scatter plots (A) between the simulated green-up dates for *Q*. *mongolica* and the start dates of the thermal growing season, and (B) between the changes in the simulated green-up dates and the start dates of the thermal growing season for each station.**

**References**

[1] Liu BH, Henderson M, Zhang YD, Xu M. Spatiotemporal change in China's climatic growing season: 1955-2000. Clim. Change. 2010; 99: 93-118. doi: 10.1007/s10584-009-9662-7.

[2] Walther A, Linderholm HW. A comparison of growing season indices for the Greater Baltic Area. Int. J. Biometeorol. 2006; 51(2): 107-118. doi: 10.1007/s00484-006-0048-5.

[3] Dong MY, Jiang Y, Zheng CT, Zhang DY. Trends in the thermal growing season throughout the Tibetan Plateau during 1960-2009. Agr. For. Meteorol. 2012; 166(15): 201-206. doi: 10.1016/j.agrformet.2012.07.013.
